# Selective Pectoralis Muscle Denervation Reduces Capsular Contracture in Sub‐Pectoral Breast Reconstruction. Long‐Term Retrospective Case‐ Control Study

**DOI:** 10.1111/ans.70371

**Published:** 2025-11-03

**Authors:** Marco Bernini, Federico Spolveri, Margherita Serra, Silvia Sordi, Cinzia Tommasi, Lorenzo Orzalesi, Lorenzo Tofani, Chiara Bellini, Diego De Benedetto, Jacopo Nori Cucchiari, Luca Visani, Carlotta Becherini, Viola Salvestrini, Lorenzo Livi, Icro Meattini

**Affiliations:** ^1^ Breast Surgery, Breast Unit, Breast Surgery Unit IRCCS Azienda Ospedaliero‐Universitaria di Bologna Bologna Italy; ^2^ Breast Surgery, Breast Unit San Jacopo Hospital Pistoia Italy; ^3^ Department of Medicine, Surgery and Neurosciences University of Siena Siena Italy; ^4^ Breast Surgery, Breast Unit, Oncology Department Careggi University Hospital Florence Italy; ^5^ Department of Statistic, Computer Science, Applications University of Florence Florence Italy; ^6^ Diagnostic Senology Unit Careggi University Hospital Florence Italy; ^7^ Radiation Oncology, Breast Unit, Oncology Department Careggi University Hospital Florence Italy

**Keywords:** animation deformity, capsular contracture, implant malrotation, implant‐based breast reconstruction, pectoralis major muscle denervation, pre‐pectoral breast reconstruction, retro‐pectoral breast reconstruction

## Abstract

**Background:**

Implant‐based breast reconstruction (IBBR) is the most common method of breast reconstruction globally. Although the traditional approach places implants in a retropectoral position, pre‐pectoral reconstruction has recently gained popularity due to its improved aesthetic outcomes and lower capsular contracture (CC) rates. However, not all patients are ideal candidates for prepectoral IBBR, making the retropectoral technique still a valuable option. Capsular contracture is a common complication of IBBR, causing discomfort, pain, poor cosmetic results, and often necessitating revision surgery, particularly in retropectoral cases. Selective denervation of the pectoralis major muscle (PMM) in retropectoral IBBR is a novel technique to reduce these complications.

**Material and Methods:**

We selected a group of denervated retro‐pec IBBR patients and compared them to non‐denervated patients. In a previous study, we analyzed the subjective opinions on the reconstruction outcomes using the BREAST‐Q postoperative questionnaire. At the same time, we compared retrospectivally the same groups, with a minimum 24‐month follow‐up, from an objective perspective, evaluating CC rates by Baker scale and possible implant malrotation through outpatient clinic visits performed by three independent breast cancer professionals.

**Results:**

The overall mean follow‐up was 3.33 (±0.47) years, and the CC rate was significantly lower in Group 1, even adjusting for the propensity score.

**Conclusions:**

PMM selective denervation has gained statistical appreciation by women and significantly reduced the CC rate from an objective evaluation in the setting of retro‐pectoral IBBR in our series.

## 1 Introduction

Implant‐based breast Reconstruction (IBBR) is the most common choice for conservative mastectomy [1, 2]. Prosthetic reconstruction can be achieved either in one stage, Direct to Implant (DTI), or in two stages using a Tissue Expander (TE). Similarly, a prosthetic device can be placed in two ways: either in a retropectoral pocket or in a prepectoral position. From the 80s [3] until a few years ago, the gold standard was to place an implant, or a TE [4], into a retropectoral pocket, but recently, with the improvement of surgical skills and with the introduction of soft tissue replacement devices, prepectoral breast reconstruction has gained worldwide success. A novel approach to this technique, using a titanium‐coated polypropylene mesh (TCPM, TiLOOOPBra, Pfm Medical, Cologne, Germany), was described for the first time in 2014 [5, 6] at our Institution. Later on, several studies have presented such an approach, mainly using acellular dermal matrices (ADMs), showing the advantages of this technique, ranging from reduction in postoperative pain to shorter hospitalization and notably improved aesthetic and functional outcomes [7].

Unfortunately, only some patients are good candidates for prepectoral breast reconstruction. Many studies have shown that strict patient selection is mandatory for the success of this type of reconstruction [6, 8]. Hence, in all those cases where a prepectoral approach is not recommended, the use of a well‐vascularized cushion, such as the Pectoralis Major Muscle (PMM) between the skin flap and prosthetic device, is still the safest option [9].

It is well known that traditional retropectoral IBBR has many drawbacks, such as animation deformity (AD), pain, discomfort, and eventually capsular contracture (CC), which may be related to the continuous movement and shearing force of the muscle itself over the prosthesis [10, 11].

CC is an excess of fibrotic and scar tissue around a breast implant [12], which clinically manifests as pain, discomfort, and unnatural firmness and distortion of the breast [13]. Traditionally, CC is evaluated by the Baker scale, which entails four classes, from first grade, where the breast results are natural, to fourth grade, which represents severe contracture with an unacceptable aesthetic outcome and/or significant patient symptoms requiring surgical intervention [14]. However, this grading system is an assessment of a single professional judgment, and currently, there is no validated and reproducible measurement of the degree of contracture [12].

Starting from our excellent results in terms of CC in pre‐pec cases [5, 6, 8], even considering the histological features of capsules [12, 15], the rationale of this present study was to investigate a possible key role of PMM in the genesis of CC and other mechanical pitfalls such as implant malrotation. The theory behind the technical innovation represented by selective denervation has been previously described in two papers [9, 10], which showed encouraging results regarding subjective aesthetic and functional outcomes in women treated with a selective PMM denervation during a retropectoral IBBR. Briefly, surgical ablation of the nerve stimulus of the PMM avoids its ceaseless action on prosthetic devices while maintaining its vascularization and viability. Retropectoral dissection to create a pocket for IBBR necessarily entails muscular detachment from bone insertions with fibers sectioned up to the fascia, thus precluding any further utility of the muscle as a mechanical tool. Therefore, as previously described, selective denervation of the PMM avoids its detrimental movement while keeping the tissue viable and thus transforming it into an autologous biological matrix. The present study aimed to objectively evaluate this procedure in the same two groups of patients previously analyzed with a subjective assessment [10], investigating the long‐term role of PMM in CC.

## 2 Materials and Methods

In 2017, a series of cases involving retropectoral IBBRs, either DTI or two‐stage IBBR, with selective PMM denervation, was started at our institution and prospectively recorded for a long‐term evaluation of the results of this procedure.

In 2020, a subjective assessment was performed utilizing the BREAST‐Q score (Memorial Sloan‐Kettering Cancer Centre and The University of British Columbia, 2006, all rights reserved) and was previously published [10]. During 2020, the routine objective evaluation of these cases was performed to compare the two groups: the case series and a series of controls submitted to the same procedures without selective PMM denervation. The selection of patients and their inclusion in the 2 groups was not randomized, but simply chosen alternatively in a timewise criterion (sequence of operative schedule date's).

Patients in Group‐1 were cases of retropectoral IBBRs either submitted to a DTI IBBR, adopting a dual plane technique using a TCPM (TiLOOOPBra, Pfm Medical, Cologne, Germany) or a two‐stage reconstruction with Tissue Expander in a complete retropectoral position. In both types of IBBR, the PMM was selectively denervated for the costal and sternal bundles while leaving the clavicular bundle intact, according to the more recent three‐nerve anatomical classification of PMM [16], as previously described [9, 10]. In the case of DTI reconstruction, the upper pole of the implant was covered by PMM, whereas the inferior surface of the implant was covered by synthetic mesh; however, in the case of TE, the coverage was entirely made by PMM.

Group 2 included patients who underwent retropectoral IBBR at the same institution and during the same period. Similarly, these cases were either DTI reconstructions or TE‐IBBR. Technical features were the same as those in Group 1, except for selective PMM denervation, which was not performed in this group of patients.

All surgical procedures for both groups were performed by the same surgeon. Identical implants—microtextured and anatomically shaped from the same manufacturer—were used for all patients. Patients in Group 1 were specifically registered in a dedicated database with baseline characteristics, oncological features, surgical details, and follow‐up data. On the days following the surgery, all patients in Group 1 were clinically evaluated by the operating surgeon using the pectoralis contraction maneuver to verify the absence of animation deformity. Group‐2 cases were extracted from the institutional database, containing all patients who underwent breast cancer surgery at our institute.

Starting in January 2023, all patients from the previous study [10] with at least a 24‐month follow‐up (from mastectomy in the case of DTI reconstructions and the second‐stage procedure in the case of TE IBBRs) were followed up at their scheduled routine outpatient visits at our institution's facilities.

Patients from both groups were visited in outpatient clinics by one breast surgeon, who did not operate on any patient in the study, one breast radiologist, and one oncology radiotherapist. The only difference from ordinary visits for this specific study was that all three professionals were asked to use the same tool when evaluating the CC grade and any possible objective and visible implant malrotation, namely the Baker four‐grade scale [13]. Each professional focuses exclusively on breast care with over ten years of experience and routinely evaluates the implant's condition during follow‐up visits. Each one provided a score for every patient; therefore, each patient received three different values for their grade of capsular contracture using the Baker classification. Scores were collected from the follow‐up visit chart records.

The study was conducted in accordance with the Declaration of Helsinki (as revised in 2013) and its later amendments. Formal consent to participate in the study was not required since an annual or semestral outpatient follow‐up visit, depending on cases, with a breast surgeon, radiologist, and oncology radiotherapist is routine practice at the Institution, with CC evaluation and possible mechanical implant displacement as well, among other parameters. Likewise, a formal Ethical Committee approval was unnecessary for the same reason.

### 2.1 Surgical Technique

**FIGURE 1:**
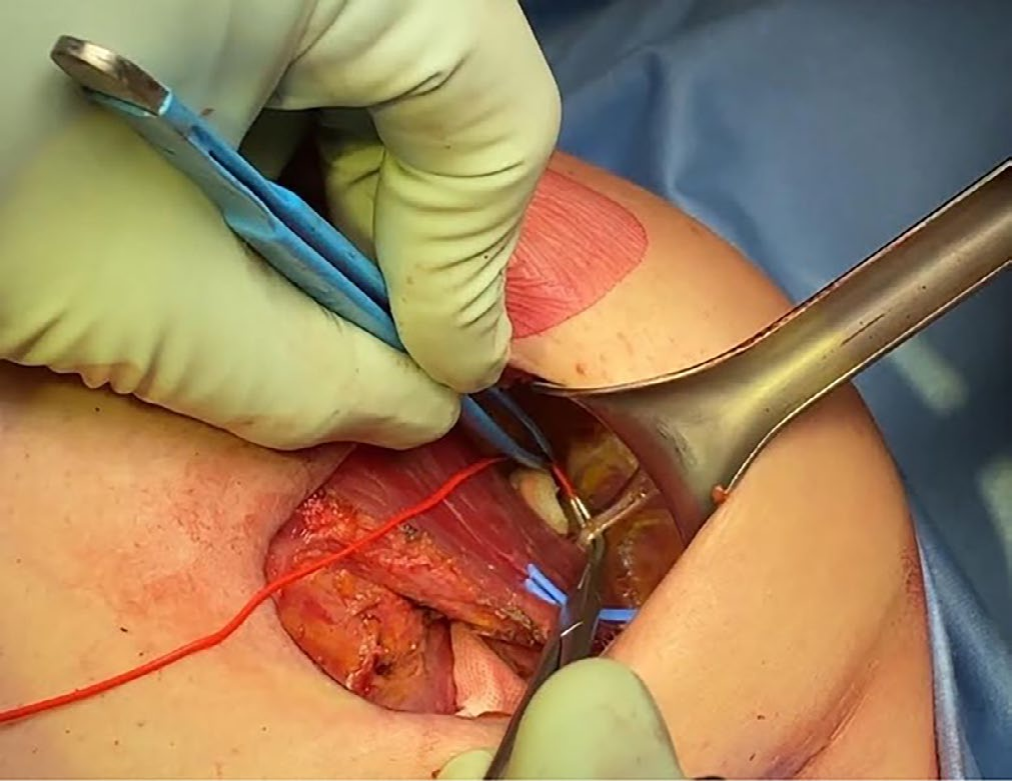
Identification of one of two middle pectoral nerve branches (MPN) through a radial outer upper quadrant incision during nipple‐sparing mastectomy.

**FIGURE 2:**
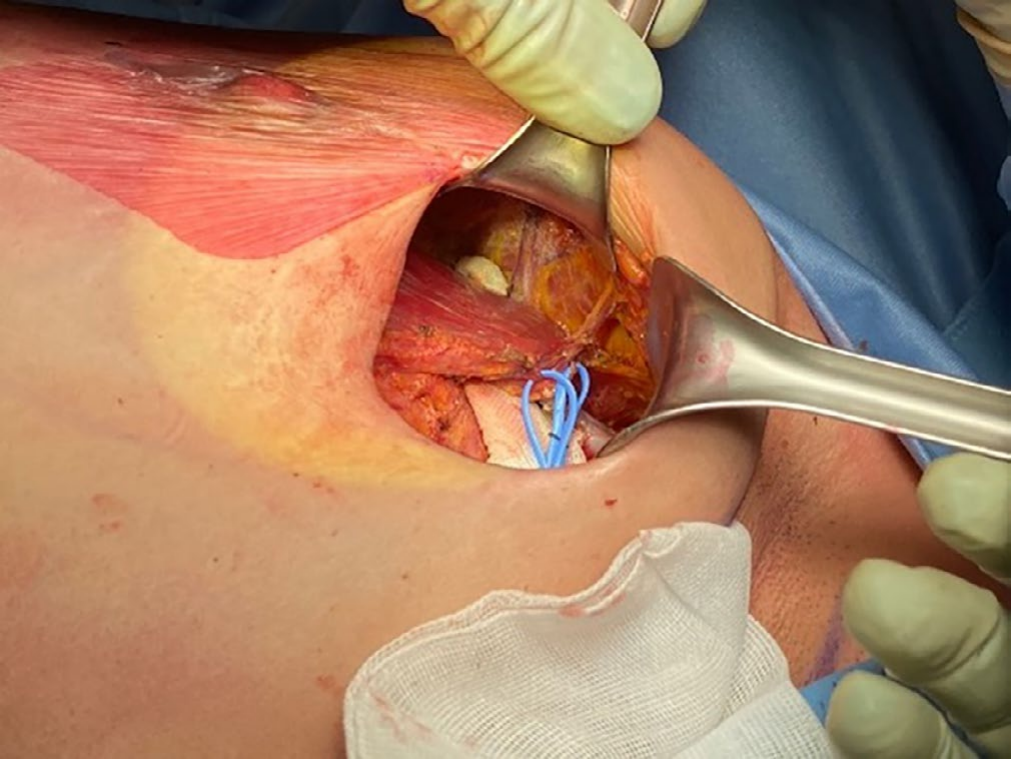
Identification of the inferior pectoral nerve (IPN), which in this case runs to the pectoralis major muscle (PMM) from the infero‐lateral border of the pectoralis minor muscle (PmM). In many cases, multiple branches of the inferior nerve are present, some of which perforate the PmM to reach the PMM.

To perform PMM selective denervation, the dissection between the pectoralis minor muscle (PmM) and PMM (the standard plane to create a submuscular pocket for retropectoral breast reconstruction) must be extended in the cephalic direction until the inferior and middle branches of the pectoral nerves are identified [9]. After exposing them, the branches can be sectioned with electrocautery or scissors (Figures 1 and 2). The adopted implants were always microtextured, and the same brand was used for every patient.

### 2.2 Statistical Analysis

Continuous variables were described as mean (±standard deviation) or median [Q1–Q3] according to variable distribution, while categorical variables were described by absolute and relative frequencies.

The Satterthwaite *t*‐test or Mann–Whitney test was used according to the Shapiro–Wilk test for normality and the F‐test for variance equality to evaluate the differences among continuous variables between the treatment groups.

Where appropriate, a Chi‐square or Fisher's exact test was used to assess the differences among categorical variables between treatment groups.

Due to the observational nature of the study, the CC grade difference between treatment groups (the primary outcome) was adjusted for propensity score using a multiple linear regression model. Malrotation was also evaluated.

A multiple logistic regression model (including the variables with *p* ≤ 0.2) with a backward selection method was used to calculate the propensity score.

A sample size estimation was not performed.

## 3 Results

All 100 patients included in the previous study, 50 in Group 1 (cases) and 50 in Group 2 (controls), were followed up, and their data were correctly retrieved according to the intended methods. The mean age of the women at the time of surgery was 49.53 (±10.90) years. The mean follow‐up was 3.33 (±0.47) years for the entire cohort, with a mean rate of CC 2.34 (±0.99).

In Group 1 (mean age 46.49 ± 10.71 years), 8 patients (16%) were smokers, 8 (16%) were former smokers, and 34 (68%) were non‐smokers. Forty‐nine patients (98%) had no comorbidities, and 9 (18%) carried a BRCA 1‐2 mutation. None of the patients clinically assessed on the days following the surgery exhibited animation deformities.

In Group 2 (mean age 52.57 ± 10.31 years), 5 patients (10%) were smokers, 6 (12%) were former smokers, and 39 (78%) were non‐smokers. Forty‐seven patients (94%) had no comorbidities, and 3 (6%) carried a BRCA 1‐2 mutation. Five patients (10%) in Group 1 and 6 (12%) in Group 2 had previously undergone radiotherapy (RT).

The majority of patients had not undergone prior surgery (80%, *n* = 40 in Group 1; 74%, *n* = 37 in Group 2), while 9 patients (18%) in Group 1 and 12 (24%) in Group 2 had previously been operated on for breast carcinoma. In Group 1, 42 (84%) nipple‐sparing mastectomies (NASM) and 8 (16%) skin‐sparing mastectomies (SSM) were performed, along with 34 (68%) sentinel lymph node biopsies (SNB) and 13 (26%) axillary node dissections (AND). In Group 2, 32 (64%) NASMs and 18 (36%) SSMs were performed, along with 29 (58%) SNBs and 18 (36%) ANDs.

For reconstruction, 39 patients (78%) in Group 1 underwent two‐stage reconstruction with tissue expanders (TE), while 11 (22%) had direct‐to‐implant (DTI) reconstruction. In Group 2, 34 patients (68%) underwent two‐stage reconstruction with TE, while 16 (32%) underwent DTI reconstruction.

Tumors were most frequently classified as pT1 (60%, *n* = 30 in Group 1; 54%, *n* = 27 in Group 2) and pN0 (68%, *n* = 34 in Group 1; 60%, *n* = 30 in Group 2).

Following mastectomy, 32 patients (65%) in Group 1 and 28 patients (65%) in Group 2 received Hormone Therapy; 13 patients (27%) in Group 1 and 12 patients (24%) in Group 2 underwent Adjuvant Chemotherapy, while 3 patients (6%) in Group 1 and 8 patients (16%) in Group 2 received post‐mastectomy radiotherapy (PMRT).

Subsequently, corrective lipofilling was necessary for 15 patients (30%) in Group 1 and 5 patients (10%) in Group 2. The baseline patient characteristics and surgical and oncological features of the two groups are shown in Table 1. We recorded statistically significant differences in age, mastectomy type, and additional fat graft procedures.

**TABLE 1:** Baseline patients‘ characteristics, surgical and oncological features.

	Group 1 (*n* = 50)	Group 2 (*n* = 50)	*p*
Age, mean (DS)	46.49 (±10.71)	52.57 (±10.31)	**0.0047**
Smoke, *n* (%)			0.5167
Active smoker	8 (16%)	5 (10%)	
Never	34 (68%)	39 (78%)	
Ex‐smoker	8 (16%)	6 (12%)	
Comorbidities, *n* (%)			0.7449
None	49 (98%)	47 (94%)	
Hypertension	1 (2%)	1 (2%)	
Diabetes	0 (0%)	2 (4%)	
BRCA1‐2 gene mutation, *n* (%)	9 (18%)	3 (6%)	0.1212
Previous breast surgery, *n* (%)			0.4592
None	40 (80%)	37 (74%)	
Augmentation/pexis	1 (2%)	1 (2%)	
Ipsilateral cancer	6 (12%)	11 (22%)	
Controlateral cancer	3 (6%)	1 (2%)	
Previous RT, *n* (%)			0.7493
Yes	5 (10%)	6 (12%)	
No	45 (90%)	44 (88%)	
Type of mastectomy, *n* (%)			**0.0226**
NASM	42 (84%)	32 (64%)	
SSM	8 (16%)	18 (36%)	
Axillary surgery, *n* (%)			0.6924
None	3 (6%)	3 (6%)	
SNB	34 (68%)	29 (58%)	
AND	13 (26%)	18 (36%)	
DTI/TE, *n* (%)			0.2601
Two‐stage with TE *n*, (%)	39 (78%)	34 (68%)	
DTI *n*, (%)	11 (22%)	16 (32%)	
pT, *n* (%)			0.3198
pT0	12 (24%)	8 (16%)	
pT1	30 (60%)	27 (54%)	
pT2	8 (16%)	12 (24%)	
pT3	0	2 (4%)	
pT4	0	1 (2%)	
pN, *n* (%)			0.8456
pN0	34 (68%)	30 (60%)	
pN1	12 (24%)	14 (28%)	
pN2	3 (6%)	5 (10%)	
pN3	1 (2%)	1 (2%)	
Chemotherapy, *n* (%)			0.1685
None	34 (69%)	30 (60%)	
Adjuvant	13 (27%)	12 (24%)	
Neo‐adjuvant	2 (4%)	8 (16%)	
PMRT, *n* (%)			0.1991
Yes	3 (6%)	8 (16%)	
No	45 (94%)	41 (84%)	
Hormone therapy, *n* (%)			0.3434
Yes	32 (65%)	28 (56%)	
No	17 (35%)	22 (44%)	
Fat graft, *n* (%)			**0.0124**
Yes	15 (30%)	5 (10%)	
No	35 (70%)	45 (90%)	

Abbreviations: AND, Axillary Node Dissection; DTI, Direct to Implant; NASM, Nipple Areola Sparing Mastectomy; PMRT, Post Mastectomy Radiation Therapy; RT, Radiation Therapy; SNB, Sentinel Node Biopsy; SSM, Skin Sparing Mastectomy; TE, Tissue Expander.

### 3.1 Primary Outcome

The CC grade, in a similar follow‐up range, was lower in Group 1 than Group 2, reaching statistical significance (*p* < 0.001) (Table 2), even after adjusting for propensity score, demonstrating that the selective PMM denervation was the only independent factor for CC rate reduction. Similarly, a significant difference emerged from the implant malrotation data, which is consistent with the rationale of the procedure, which is to avoid any possible movement of a finely reconstructed breast.

**TABLE 2:** Time of follow‐up, grade of capsular contracture (CC), and number of implant malrotation.

	Group 1 (*n* = 50)	Group 2 (*n* = 50)	*p*
Follow‐up, median, months	40.56	39.36	0.2795
Grade of CC, median (range)	1.33 (1–3)	3.33 (1.66–4)	**< 0.001**
Implant malrotation	0	6	0.0267

Upon further examination of the two groups, we identified significant associations between Group 1 and the type of mastectomy (NASM 84%, *p* < 0.05), in addition to notable correlations between Group 1 and secondary fat graft (30%, *p* < 0.05).

## 4 Discussion

Implant‐based breast reconstruction is the most widely used technique for breast reconstruction worldwide [2, 17].

The retropectoral approach has been the gold standard for more than two decades in the breast reconstructive setting [4, 18] because of its well‐known advantages in terms of complications compared to the former subcutaneous reconstructions attempted in the ‘70s [3, 19]. On the other hand, the retro‐pec procedure resulted in other drawbacks and complications such as AD, window‐shading effect, insufficient lower pole fulness, postoperative pain, discomfort, and CC [20, 21, 22]. Such side effects have also been described in aesthetic dual‐plane breast augmentation [23].

The prepectoral approach was back in vogue with the introduction of new surgical materials (TPCM and ADMs) as possible replacements for the muscular pocket [6, 7, 23, 24]. Several studies have shown safety and good aesthetic results with this technique, both in DTI IBBR and two‐stage TE IBBR [4, 5, 6, 8, 25], with a CC rate ranging from 0% to 5%, no AD, and excellent aesthetic results [5, 26, 27]. Unfortunately, not all patients are good candidates for prepectoral IBBR, and in these cases, the retropectoral technique, either dual‐plane or completely retropectoral, remains a sound option [9, 10, 18].

Sobti et al. [28] reported that the CC rate was higher in patients who underwent a retro‐pectoral IBBR than in those who underwent prepectoral IBBR (53.7% vs. 30%). The rate of CC increases fourfold in patients who have undergone post‐ mastectomy radiation therapy (PMRT).

Larsen et al. [12] reported that CC affects between 2.8% and 20.4% of women with breast implants and that its etiology remains undetermined. However, multiple clinical risk factors, including infection, PMRT, implant surface texture, and implant position, have been suggested as possible causes.

Sinnott et al. [29] reported a rate of CC in retro‐pectoral IBBR of 2.9%, which increased to 52.2% when patients underwent PMRT and showed that the contracture rate was three times higher for the retro‐pectoral patients with PMRT than for the pre‐pectoral patients with PMRT (52.2% vs. 16.1%).

In a recent study by King et al. [26], the CC rates were higher in retropectoral patients than in the prepectoral group (9.6% vs. 4.9%). The prepectoral group also had significantly decreased AD compared to the retropectoral group (0% vs. 19.7%).

A recent meta‐analysis by Li et al. [4] compared prepectoral and retropectoral IBBR (DTI and TE). Once again, a lower CC rate was found in the prepectoral group (OR 0.16, 95% CI 0.05, 0.53), and CC remained the most reported complication and reason for reoperation. They speculate this might be due to a significantly thinner capsule wall in pre‐ pec cases and no mechanical stress over the implant. They also demonstrated that the incidence of CC was similar between ADMs and TCPM cases.

Therefore, it is correct to assume, or at least hypothesize, that the muscle may be a critical point in the genesis of CC [18], which is also supported by the fact that AD, which is related to the muscle and one of the main drawbacks of the retropectoral IBBR, has never been reported in the pre‐pectoral setting. AD is the lateral displacement of the implant whenever the PMM contracts and creates a visible distortion of the breast contour and a sprain of the nipple‐areola complex. This complication is often underestimated, but it may be the cause of severe psychological discomfort in patients [10, 11, 30]. The overall incidence of AD is 75.6% in patients undergoing breast reconstruction and 77.5% after retropectoral breast augmentation [31, 32].

Another study by Becker and Fregosi [33] concluded that AD is an inevitable consequence of submuscular implant placement, grading the severity of this complication on a scale of 4, where grade I corresponds to minimal breast distortion and grade IV to severe distortion.

AD is usually corrected by repositioning the implant to a prepectoral position [32]. It is also described that the injection of neuromodulator drugs, like botulinum toxin, can paralyze the muscle and relieve symptoms, but this is temporary and may expose the implant to risks of injury [34].

Moreover, Sobti et al. [28] reported that patients with retropectoral IBBR and PMRT who underwent revision surgery for CC had an impressive release of their contracted breast and an upper pole drop of approximately 2–4 cm after general anesthesia induction with muscular relaxation. This is an evident clinical sign that the muscle is a leading factor in the contracture.

All these findings were the rationale for our hypothesis that AD and CC are two sides of the same coin, represented by the PMM contraction over the implant, and the rationale for introducing selective denervation of the PMM to avoid these drawbacks [18]. The modification of the standard technique in the retropectoral approach was described in our previous study for the first time [9]. In 2018, Eck et al. [35] described the pectoralis nerve ablation by selective bipolar electrocautery not associated with implant exchange in a patient with AD after bilateral retropectoral IBBR, who was then free from AD at a 2‐year follow‐up. Similarly, a study by Casella et al. [11], on quite a large number of patients (62), showed excellent results with pectoral nerve neurotomy in terms of AD disappearance. Recently, we published the result of a long‐term analysis of the subjective opinion of women who underwent this procedure, evaluated using the BREAST‐Q postoperative questionnaire, and compared it with a group of patients who underwent a classic procedure without selective PMM denervation. Our results showed a better outcome in terms of quality of life in the case series subjected to PMM denervation, with a statistically significant difference [10]. In the present study, we provided a further analysis of the same two groups, but in terms of objective evaluation, to investigate the CC rate.

The results highlighted a significantly higher CC rate in the control group, and the propensity score confirmed that denervation was the only independent factor related to a lower CC. These findings corroborate our initial hypothesis that the muscle is the main culprit in the genesis of CC. Similarly, our data showed that the muscle might be responsible for another late complication, implant malrotation, which was never recorded in our limited series of denervated patients. Implant displacement is a relatively common late complication of IBBR, with a very different rate from that reported in previous studies [36, 37, 38].

The mean follow‐up duration was adequate over three years, with no difference between the two groups. There was, by the way, a significant difference in terms of age (denervated patients are younger), type of mastectomy (nipple‐sparing mastectomy was higher in Group 1), and fat graft procedures (more numerous in the denervated group) between cases and controls; nonetheless, the propensity score test didn't show any confounding factors to explain the difference in CC rate.

The higher number of secondary fat grafts in Group 1 may be explained by the physiological thinning of the pectoralis major muscle that can occur following denervation, as described in our previous study [9]. We believe that this minor aesthetic drawback is offset by the objective advantage of a lower incidence of capsular contracture compared to the traditional technique and the subjective benefit reflected in patient questionnaire scores, indicating a preference for this procedure [10].

## 5 Limitations

We should consider the limited number of samples analyzed in our study, only 50 cases per group, and the lack of extended follow‐up (mean 3.35 years). Nevertheless, our results are consistent with our previous subjective evaluation, and the literature shows a significant difference in terms of CC between prepectoral and retropectoral cases, with a prominent link to muscular activity over the implant, as we investigated by selectively paralyzing the muscle during retropectoral procedures.

Another significant limitation of this study is the method used to evaluate the extent of CC. The Baker classification of Capsular Contracture is currently the most widely used in the literature and is generally accepted [13, 39, 40, 41], but it lacks interrater reliability [42]. It would be beneficial if a validated instrument recognized by the scientific community could be employed to provide more objective and reproducible values.

## 6 Conclusion

In conclusion, selective PMM denervation represents an intriguing technical modification of the standard retropectoral approach. It shows a significant reduction in CC and implant displacement rates compared to the standard technique and, therefore, represents a valid alternative to a prepectoral reconstruction whenever such a procedure is not indicated.

Certainly, in the study there is a selection bias with differences between the two groups: these data suggest lower capsular contracture rates but this would need to be verified in a randomised controlled trial to be conclusive.

## Author Contributions

**Marco Bernini:** supervision, validation, conceptualization, investigation, writing – original draft. **Silvia Sordi:** visualization, validation, and data curation. **Federico Spolveri:** supervision, writing – review and editing. **Cinzia Tommasi:** validation, conceptualization, investigation, data curation. **Lorenzo Orzalesi:** validation, supervision, resources. **Margherita Serra:** supervision, resources, corresponding author. **Lorenzo Tofani:** formal analysis, software. **Chiara Bellini:** supervision, resources. **Diego De Benedetto:** supervision, resources. **Jacopo Nori Cucchiari:** supervision, validation, resources. **Luca Visani:** supervision, resources. **Carlotta Becherini:** supervision, resources. **Viola Salvestrini:** supervision, resources. **Lorenzo Livi:** supervision, validation, project administration. **Icro Meattini:** supervision, validation, visualization, project, administration, resources, data curation.

## Conflicts of Interest

The authors declare no conflicts of interest.

## Data Availability

The data of this study are available from the corresponding author upon reasonable request to the corresponding author at: https://zenodo.org/records/15876884.
